# New Applications of High-Resolution Analytical Methods to Study Trace Organic Compounds in Extraterrestrial Materials

**DOI:** 10.3390/life9030062

**Published:** 2019-07-26

**Authors:** Hiroshi Naraoka, Minako Hashiguchi, Yu Sato, Kenji Hamase

**Affiliations:** 1 Research Center for Planetary Trace Organic Compounds, Kyushu University, 744 Motooka, Nishi-ku, Fukuoka 819-0395, Japan; 2 Department of Earth and Planetary Sciences, Kyushu University, 744 Motooka, Nishi-ku, Fukuoka 819-0395, Japan; 3 Graduate School of Pharmaceutical Sciences, Kyushu University, 3-1-1 Maidashi, Higashi-ku, Fukuoka 812-8582, Japan

**Keywords:** extraterrestrial materials, high mass resolution, high-resolution chromatography, in situ analysis, molecular imaging, enantiomeric amino acids

## Abstract

Organic compounds are present as complex mixtures in extraterrestrial materials including meteorites, which may have played important roles in the origin of life on the primitive Earth. However, the distribution and formation mechanisms of meteoritic organic compounds are not well understood, because conventional analytical methods have limited resolution and sensitivity to resolve their molecular complexity. In this study, advanced instrumental development and analyses are proposed in order to study the trace organic compounds of extraterrestrial materials: (1) a clean room environment to avoid organic contamination during analysis; (2) high-mass-resolution analysis (up to ~150,000 *m/Δm*) coupled with high-performance liquid chromatography (HPLC) in order to determine the elemental composition using exact mass for inferring the chemical structure; (3) superior chromatographic separation using a two-dimensional system in order to determine the structural and optical isomers of amino acids; and (4) in situ organic compound analysis and molecular imaging of the sample surface. This approach revealed a higher complexity of organic compounds with a heterogeneous distribution in meteorites. These new methods can be applied to study the chemical evolution of meteoritic organic compounds as well as the molecular occurrence in very-low-mass extraterrestrial materials such as asteroid-returned samples.

## 1. Introduction

Organic compounds are distributed widely in natural environments, and are characterized by their diverse chemical structures. The occurrence of organic molecules in astrobiological samples such as meteorites is critical to understand the chemical evolution and biological activity in the universe. In particular, primitive asteroids and comets may have contributed to the delivery of organic matter and water to the primitive Earth. In 2010, the Hayabusa spacecraft successfully returned to Earth with surface grains from asteroid 25143 Itokawa, and this asteroid-return sample has allowed for the investigation of a direct relationship between asteroids and meteorites. Based on chemical and isotopic analyses, the grains of Itokawa are equivalent to LL5-6 ordinary chondrite [1,2]. However, although the water content of the Itokawa grains has been reported [3], no indigenous organic matter has yet been discovered from the Itokawa samples [4]. In 2018, the Hayabusa2 spacecraft reached asteroid Ryugu (a C-type asteroid, carbonaceous meteorite equivalent), to collect surface samples, which will be delivered to Earth in late 2020.

Organic compounds in geochemical samples are frequently analyzed by mass spectrometry coupled with chromatography. However, extraterrestrial organic compounds are typically present in very low concentrations, yet occur as very complex mixtures. Compounding this problem, the quantity of material obtained by sample-return missions from asteroids is extremely limited. Hence, in order to obtain detailed information concerning asteroidal organic compounds, the development of ultra-high-sensitivity and resolution analyses is required. As terrestrial organic contaminants are common in routine laboratory conditions, analytical procedures must be performed under clean conditions to avoid terrestrial contamination. Moreover, non-destructive organic analysis is preferred in order to preserve precious sample material for possible future additional study.

The Research Center for Planetary Trace Organic Compounds (PTOC) of Kyushu University is dedicated to the study of trace organic compounds under clean conditions. In this article, we will introduce the development of analytical methods for meteoritic organic compounds at the PTOC Center: (1) clean room technology; (2) high-mass-resolution mass spectrometry; (3) superior chromatographic separation; and (4) in situ organic compound analysis with molecular imaging.

## 2. Equipment

### 2.1. Clean Environment

Clean room technology is essential to the study of trace organic compounds in extraterrestrial materials in order to avoid terrestrial contamination, and to reduce analytical background. In the case of interplanetary dust particles and the asteroid-return samples from Itokawa, grains smaller than 100 μm are common [5], and are generally handled using a micro-manipulator in a clean chamber [6]. The PTOC center has two clean rooms (both ISO Class 6) for sample preparation and mass spectral analysis with an air shower unit. Both rooms are constructed of stainless steel (SS 304 grade) bearing four-layer filters (acid, alkaline, organic chemical, and high-efficiency particulate air (HEPA)) filters for air cleaning. The average number of dust particles smaller than 1.0 μm and 0.3 μm in diameter were approximately 4600 and 8100 counts/m^3^ of the laboratory atmosphere, respectively, as measured by a particle counter (METONE HHPC+, Beckman Coulter). A clean bench (SS 304 grade, ISO Class 5) is installed in the preparation room. The clean bench also has four-layer filters for air cleaning, and no particles were detected inside the clean bench (~0 counts for both 1.0 μm and 0.3 μm particles) by the counter. An optical microscope inside the clean bench was used to handle samples for single-grain or desorption electrospray ionization/mass spectrometry (DESI/MS) analysis (see below).

### 2.2. High-Resolution Mass Spectrometry

Fourier transform ion cyclotron resonance mass spectrometry (FT-ICR/MS) facilitated ultra-high-resolution MS to measure the exact mass of molecular ions with a mass resolution of up to a few million (*m/Δm*). Under such high mass resolution, it is necessary to consider the mass of electrons for the assignment of molecular ions. This ultra-high-resolution MS provides a sufficiently highly resolved mass determination that allows for the assignment of a molecular formula with a high degree of confidence. In the various solvent extracts of the Murchison meteorite, tens of thousands of different mass peaks consisting of C, H, N, O, and/or S were observed, in which >14,000 unique molecular formulae were assigned to CHO, CHOS, CHNO, and CHNOS compositions by FT-ICR/MS using negative ions with electrospray ionization (ESI) [7,8]. In addition, new Mg-containing organics (MgCHO compounds) were discovered in the solvent extract of various meteorites [9]. Nevertheless, the specific organic compounds currently identified in meteorites probably correspond to less than 1% of the total number of compounds present, as a single molecular formula may correspond to multiple chemical structures (i.e., structural and optical isomers).

In order to fully reveal the complete molecular diversity, it is necessary to perform gas (GC) or liquid chromatography (LC) using appropriate column(s) depending on the molecular structure and possible optical isomers. For example, using high-performance liquid chromatography (HPLC)–MS with mass resolution of ~100,000 (*m/Δm*) at *m/z* 400, more than 600 C*_n_*H*_m_*N and C*_n_*H*_m_*N_2_ compounds were identified in the methanol extract of the Murchison meteorite [10]. The molecular structures were identified as alkylated pyridine (C*_n_*H_2*n*−5_N) and imidazole (C*_n_*H_2*n*−2_N_2_) homologues by comparison with retention times for typical standards, in conjunction with MS/MS analysis. The alkylpyridine and alkylimidazole homologues were predominant in the CM chondrites (Murchison and Murray), while alkylpiperidine homologues (C_n_H_2n+1_N) were predominant in the Yamato 00259 CR meteorite [11], and this difference in molecular distribution between the CM and CR chondrites has been explained by different redox conditions on the meteorite parent bodies.

### 2.3. High Separation Efficiency Chromatography

Meteoritic organic compounds are present not only as significantly complex mixtures but also at extremely low concentrations for each compound. Hence, high-sensitivity high-resolution chromatography is essential in order to identify and quantify such complex and trace organic compounds. Recent chromatographic techniques such as nanoLC and multi-dimensional chromatography achieve high sensitivity and chromatographic separation. For example, nanoLC equipped with nanoESI can enhance the sensitivity of detection by three orders of magnitude compared to that of conventional HPLC, and hence greatly minimizes the sample amount requirement.

Multi-dimensional chromatography can promote the enhanced separation of organic compounds using more than two columns in different modes (e.g., reversed phase, stereoselective phase), which can also reduce the background contribution and therefore improve analytical sensitivity. A two-dimensional chiral high-performance liquid chromatographic (2D-HPLC) system has been established for the analysis of extraterrestrial amino acids using high-sensitivity fluorescence detection [12], where a C18 column was used as the first chromatography column and an enantioselective Pirkle-type column was adapted as the second chromatography column for the separation of DL-amino acids.

### 2.4. Organic Compound Imaging by in Situ Analysis

Meteoritic organic compounds have generally been analyzed using solvent extracts of powdered samples, wherein the spatial distribution of organic compounds within the meteorite sample cannot be determined. As the petrological texture of chondritic meteorites is heterogeneous, any specific organic–mineral associations would suggest that the distribution and occurrence of organic compounds in meteorites might also be heterogeneous. To date, in situ analysis of meteoritic organic compounds has rarely been performed. Volatile compounds such as polycyclic aromatic hydrocarbons (PAHs) have been analyzed using the so-called micro two-step laser mass spectrometry (μL^2^MS) [13,14] or time-of-flight secondary ion mass spectrometry (ToF-SIMS) [15] with a few to tens of micrometers resolution. These analyses have been carried out in vacuo using laser ablation or ion sputtering for ionization, which can damage the sample surface.

Desorption electrospray ionization (DESI) is a gentle ionization method performed under ambient atmosphere conditions [16]. Using a spray of electrically charged solvent such as methanol, soluble organic compounds are desorbed and ionized from the sample for introduction to a mass spectrometer, with a typical surface spatial resolution of 50–100 μm. Molecular imaging of the surface of the Murray and Murchison meteorites were performed by DESI/HRMS, and various alkylated CHN homologous compounds were identified [17,18]. These CHN compounds were distributed heterogeneously in the matrices of both meteorites. In the case of the Murray meteorite, each homologous series (e.g., C*_n_*H_2*n*−5_N (alkylpyridines) or C*_n_*H_2*n*−2_N_2_ (alkylimidazoles)) showed the same spatial distribution—that is, all of the C*_n_*H_2*n*−5_N compounds were co-located with the other C*_n_*H_2*n*−5_N compounds, and all of the C*_n_*H_2*n*−2_N_2_ compounds were co-located with the other C*_n_*H_2*n*−2_N_2_ compounds. However, the alkylpyridines and alkylimidazoles occurred separately in different locations (i.e., the C*_n_*H_2*n*−5_N compounds and the C*_n_*H_2*n*−2_N_2_ compounds were not co-located [17]), suggesting a different source for each CHN compound series or a possible chromatographic effect associated with fluid movement in the meteorite parent body. In a previous study of the Murchison meteorite [18], only the alkylimidazole series was identified.

## 3. Sample and Analytical Procedures

### 3.1. Sample

In this study, soluble organic matter (SOM) was analyzed using a minimal amount of the Murchison meteorite by the following: (1) nanoLC/HRMS analysis of the methanol extract; (2) enantiomer separation of chiral amino acids by 2D-HPLC; and (3) organic compound imaging by DESI/HRMS. The sample preparation was performed in the clean room of PTOC. The organic solvents (methanol, acetonitrile, and HCOOH) used were LC/MS-grade, purchased from Wako Chemicals. All glassware used in this study were baked at 500 °C for 3 h prior to use.

### 3.2. NanoLC/HRMS

**Figure 1:**
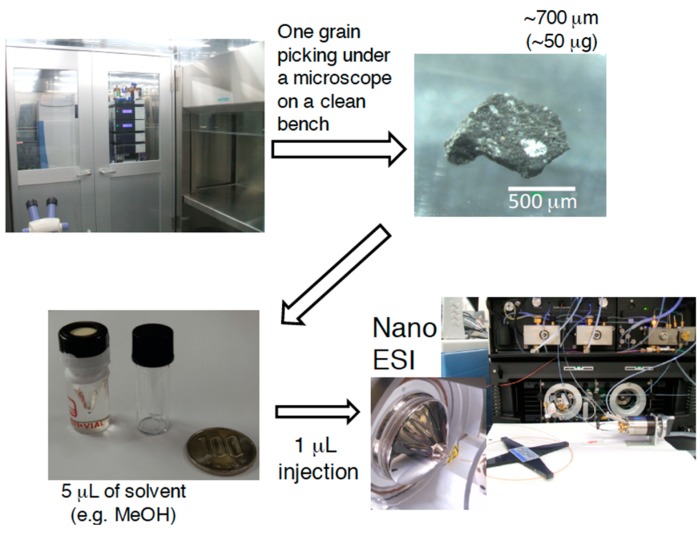
One grain analysis for the methanol extract of the Murchison meteorite by nanoLC-nanoESI/HRMS.

One grain (~500 μm in diameter, ~50 μg) of the Murchison meteorite was obtained by chipping on a clean bench and put into a 300-μL V-shape bottom glass vial (Figure 1). Five microliters of methanol was added and subjected to ultrasonic extraction (5 min). One microliter of sample solution was injected into the nanoLC (UltiMate 3000 RSLCnano) coupled with a hybrid quadrupole-Orbitrap mass spectrometer (Q-Exactive Plus) equipped with a nanoelectrospray ion source. A narrow reverse-phase column (Acclaim PepMap C18, 75 μm i.d. × 15 cm length) was used with a program of eluent mixture of A (H_2_O with 0.1% HCOOH) and B (CH_3_CN with 0.1% HCOOH) at 200 nL/min, where the A/B ratio was changed by a linear gradient from 99/1 to 1/99 in 30 min, then maintained at a 1/99 ratio for 20 min. The electric voltage of nanoESI and the temperature of the ion transfer tube were set to 3.0 kV and 280 °C, respectively. Positive mass spectra were taken by full scan over a range of *m/z* 62–600 with a mass resolution of *m/Δm* ~140,000 at *m/z* 200.

### 3.3. Amino Acid Analysis by 2D-HPLC

**Figure 2:**
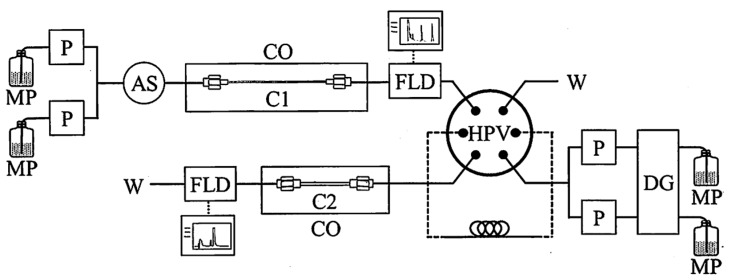
Schematic flow diagram of 2D-HPLC for enantiomeric analysis of trace amino acids. MP, mobile phase; P, pump; AS, auto sampler; CO, column oven; FLD, fluorescence detector; HPV, high-pressure valve; W, waste. C1: Column 1, Capcell Pak C18 ACR S3 (1.5 mm i.d. × 1000 mm, 45 °C); C2: Column 2, KSAACSP-001S (1.5 mm i.d. × 250 mm, 25 °C).

A powder sample (2.11 mg) and four grains ranging from 29 to 128 μg were prepared from a single specimen of the Murchison meteorite. The powder sample was used to establish the chromatographic conditions of 2D-HPLC according to the method of Hamase et al. [12]. Each grain was put into a 1-mL V-shape bottom glass ampoule. After 100 μL of doubly distilled HCl was added to each ampoule, the glass ampoules were sealed by flame and heated at 105 °C for 20 h. The reaction products were dried in vacuo using a diaphragm pump with a liquid N_2_ trap, and neutralized with aqueous NaOH solution. An aliquot of this solution was placed in a light-shielded vial followed by the addition of Na-phosphate buffer (pH = 9). For high-sensitivity fluorescence detection, 4-fluoro-7-nitro-2,1,3-benzoxadiazole (NBD-F) in CH_3_CN (200 mM) was added and the vial was heated at 60 °C for 2 min. The derivatized amino acids in an aqueous 2% (*v*/*v*) trifluoroacetic acid solution were applied to the 2D-HPLC system (Figure 2) using a reverse phase column (Capcell Pak C18 ACR S3) as the first dimension. The target fractions were automatically introduced into the second dimension and further separated by a Pirkle-type enantioselective column (KSAACSP-001S). The detailed analytical conditions were described elsewhere [12].

### 3.4. DESI/HRMS Analysis

**Figure 3:**
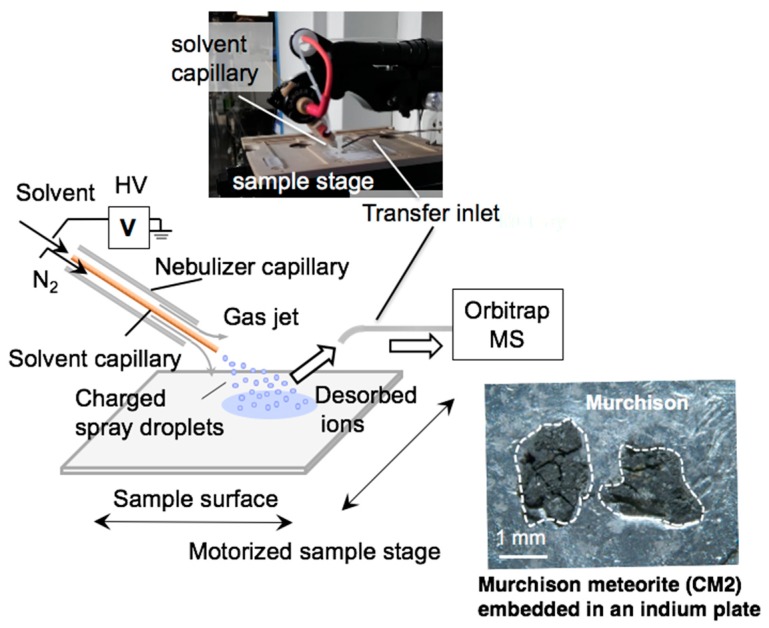
Molecular imaging on the surface of the Murchison meteorite by desorption electrospray ionization/high-resolution mass spectrometry (DESI/HRMS) under ambient conditions in the clean room of the Research Center for Planetary Trace Organic Compounds (PTOC). HV denotes high voltage.

Two fragments of the Murchison meteorite (a few millimeters in size) were embedded in an indium plate (Figure 3). The DESI/HRMS imaging was performed using a 2D DESI ion source (Omni Spray Source 2D) coupled with a hybrid quadrupole-Orbitrap mass spectrometer (Q-Exactive Plus) in the clean room of PTOC. CH_3_OH (100%) was sprayed onto the sample at a rate of 2 μL/min. The electrospray voltage was set to 3 kV, and the nebulizer N_2_ gas pressure was ~100 psi. DESI images were acquired by continuously moving the surface beneath the spray at a constant velocity. Positive ions ranging from *m/z* 70 to 750 were collected in a full scan mode with a mass resolution of approximately 140,000 (*m/Δm* at *m/z* = 200). The detailed analytical procedure has been described elsewhere [18].

## 4. Results and Discussion

### 4.1. NanoLC/HRMS

**Figure 4:**
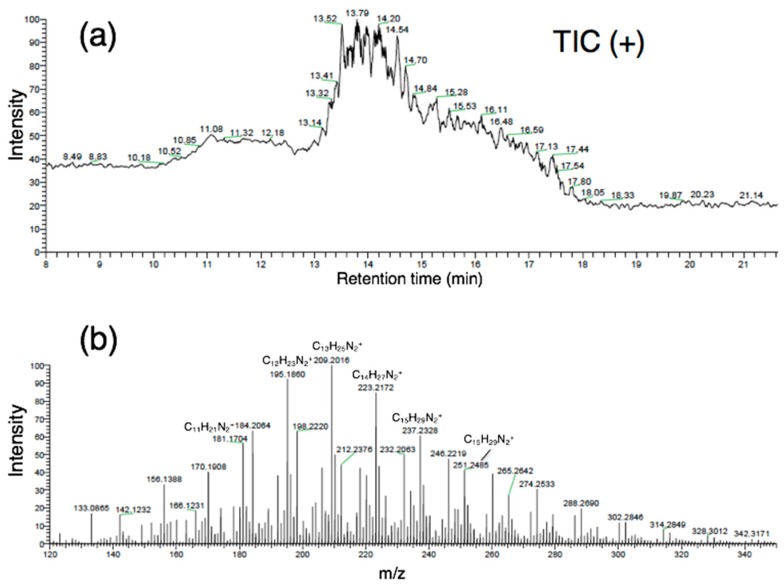
(**a**) Total ion current (TIC) chromatogram and (**b**) its mass spectrum obtained from one grain (~50 μg) of the Murchison meteorite by nanoLC/HRMS.

The total ion current (TIC) chromatogram of nanoLC/HRMS analysis with its mass spectrum is shown in Figure 4. Many of the intense peaks were assigned to C*_n_*H*_m_*N_2_^+^ and C*_n_*H*_m_*N^+^ chemical compositions within the mass precision of 5 ppm. In particular, the C*_n_*H_2*n*−1_N_2_^+^ and C*_n_*H_2*n*−6_N^+^ series were predominant, and their original chemical compositions were C*_n_*H_2*n*−2_N_2_ and C*_n_*H_2*n*−7_N, respectively, given the protonation during ionization, which would be assigned to alkylated imidazoles and pyridines, respectively. The occurrence of these CHN homologous compounds were very similar to that of the Murchison extract in a previous study using conventional HPLC/HRMS with ESI [10]. Approximately 800 mg of powder of the Murchison meteorite was used in the previous study [10], but this study used only ~50 μg of sample grain (i.e., more than four orders of magnitude less sample material). The single small grain of sample was directly soaked in a very small volume of methanol (5 μL), and the simple preparation procedure required less time. However, the nanoLC/HRMS signal intensity showed more fluctuation compared to the conventional HPLC/HRMS analysis due to the lower nanoLC/HRMS signal intensity. Moreover, the mass precision (typically up to 5 ppm) of this study was a little lower than that of the previous conventional HPLC/HRMS analysis (approx. 1 ppm) [10].

**Figure 5:**
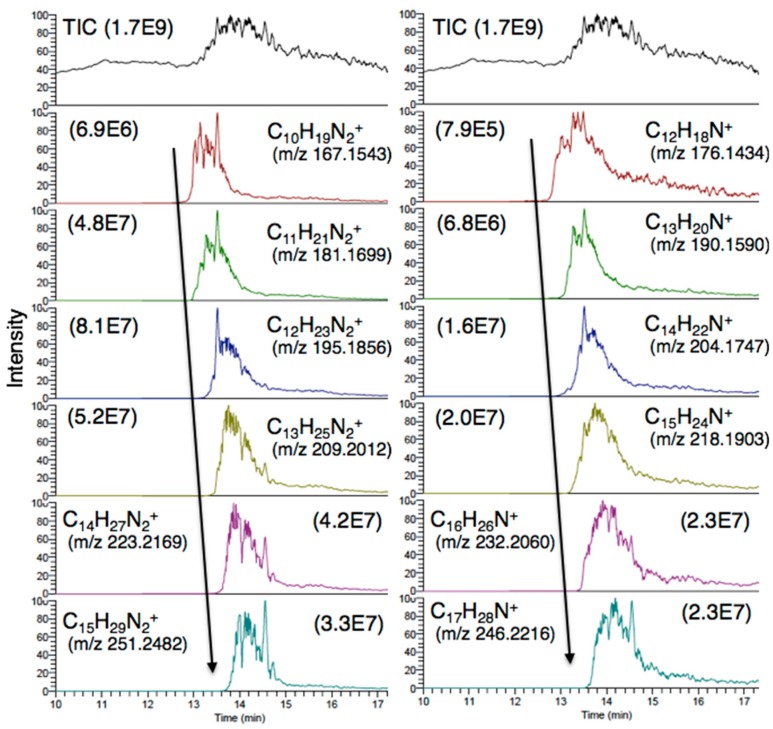
Mass chromatograms of C*_n_*H_2*n*−1_N_2_^+^ series (**left**) and C*_n_*H_2*n*−6_N^+^ series (**right**) by nanoLC/HRMS of the methanol extract from one grain (~50 μg) of the Murchison meteorite. Numbers in parentheses denote the intensity of the total ion current (TIC) for each ion (*m/z*). Note that the longer alkyl-chain homologues exhibited longer retention times with increasing hydrophobicity using reverse-phase C18 column chromatography.

The mass chromatograms of the alkylated homologues of C*_n_*H_2*n*−1_N_2_^+^ and C*_n_*H_2*n*−6_N^+^ series are shown in Figure 5. Using reverse-phase C18 column chromatography, the homologous compounds showed regular chromatographic shifts, where the longer alkyl-chain homologues exhibited longer retention times with increasing hydrophobicity. This systematic chromatographic shift indicates that the core structures (imidazole and pyridine) were identical for these homologues with different alkyl chain lengths. Moreover, the alkylimidazoles were more abundant than the alkylpyridines in this study of the Murchison meteorite (Figure 4), in contrast to the previous study which found that the alkylpyridines were more abundant than the alkylimidazoles [10]. The different relative abundance of alkylpyridines vs. alkylimidazoles for differently-sized sub-samples both taken from the Murchison meteorite implies a heterogeneity of both the absolute abundances of CHN compounds, and also a heterogeneity of the relative abundances of CHN compounds on a small scale of sampling (confirmed by DESI/HRMS analysis, below).

### 4.2. Enantiomer Distribution of Amino Acids

**Figure 6:**
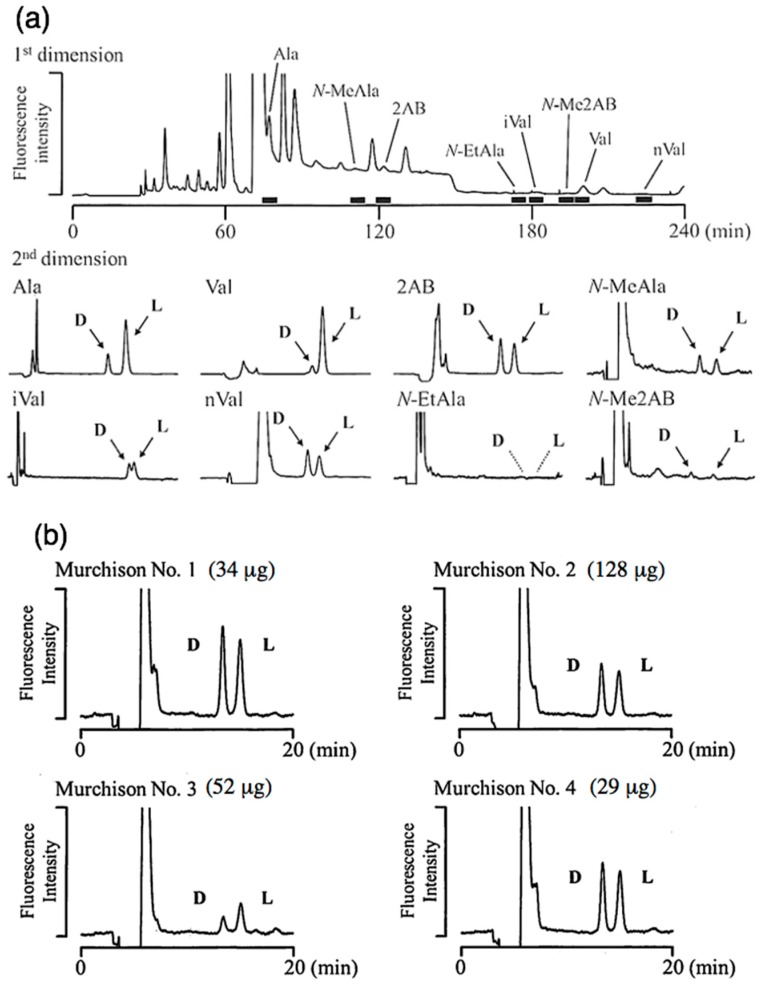
2D-HPLC separation of amino acid enantiomers in the Murchison meteorite by high-sensitivity fluorescence detection. (**a**) Chromatograms of eight enantiomeric amino acids using 2.11 mg of powder sample, and (**b**) the enantiomeric distribution of 2-amino butyric acid (2AB) from the four fragments (34–128 μg).

Eight chiral amino acids were chosen as targets in this study: alanine (Ala), valine (Val), 2-aminobutyric acid (2AB), norvaline (nVal), *N*-methylalanine (*N*-MeAla), isovaline (iVal), *N*-ethylalanine (*N*-EtAla), and *N*-methy-2-aminobutyric acid (*N*-Me2AB). In both powder and grain samples, proteinogenic amino acids (Ala and Val) showed a strong L-enrichment, which is similar to the recent result measured by GC/MS [19], and it is likely that the L-rich proteinogenic amino acids were contributed from terrestrial contamination prior to the analysis. In contrast, most non-proteinogenic amino acids showed an almost 50/50 ratio of D/L enantiomeric distribution, with 2AB being relatively abundant and clearly present as racemic mixtures (Figure 6).

The grain-level amino acid analysis showed heterogeneous concentrations of 2AB (4 to 13 nmol/g). Although three grains (Nos. 1, 2, and 4) yielded close to a 50/50 D/L ratio of 2AB, one sample (No. 3) showed L enrichment (Figure 6). This result may suggest a heterogeneous distribution of 2AB enantiomers in the Murchison meteorite, and is consistent with previously reported results [20,21,22]. Further detailed examination is needed to confirm the possible spatial distribution of the enantiomeric excess in extraterrestrial materials, and will require analytical methods such as those developed in this study which can facilitate examination of the enantiomer distribution using microgram-sized samples.

### 4.3. DESI/HRMS Analysis

**Figure 7:**
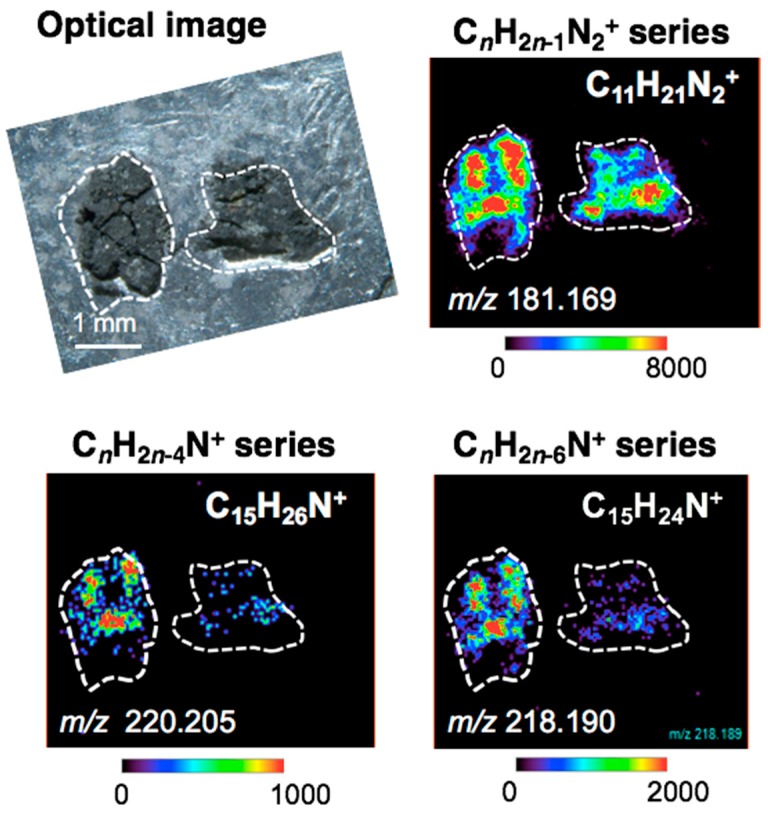
Spatial distribution of CHN compounds in the Murchison meteorite revealed by DESI/HRMS. The spatial distribution of C*_n_*H_2*n*−1_N_2_^+^, C*_n_*H_2*n*−4_N^+^, and C*_n_*H_2*n*−6_N^+^—corresponding to alkylimidazoles, unsaturated-, and saturated-alkylated pyridines, respectively—is similar. Color bars show ion intensity (note that scales are different for the three CHN series).

The spatial distribution of some CHN compounds in the Murchison meteorite measured by DESI/HRMS is shown in Figure 7. The alkylated homologues of the C*_n_*H_2*n*−1_N_2_^+^ (alkylimidazole) and C*_n_*H_2*n*−6_N^+^ (alkylpyridine) series were the most abundant CHN compounds, consistent with the nanoLC/HRMS analyses. These CHN compounds were observed to be distributed heterogeneously in the matrix, and all of the homologues in each homologous CHN series had the same spatial distribution—that is, all of the alkylimidazole compounds were co-located with the other alkylimidazole compounds, and all of the alkylpyridine compounds were co-located with the other alkylpyridine compounds. This study of the Murchison meteorite found that the alkylimidazole and alkylpyridine compounds were co-located with each other (i.e., these two homologous CHN series were co-located). This is in contrast with a previous DESI/HRMS study of the Murray meteorite, which found that the alkylimidazole and alkylpyridine compounds occurred in different locations—that is, these two homologous CHN series were not co-located [17]. Furthermore, a previous DESI/HRMS study of the Murchison meteorite found only alkylimidazole compounds, and no alkylpyridine compounds were detected [18].

These different spatial distributions for the different CHN homologous series in the Murray and Murchison meteorites might be attributed to differential fluid activity associated with minerals in the matrix or to different reaction processes for the CHN compounds on the parent bodies. Further DESI/HRMS chemical imaging will improve our understanding of organic compound distributions with respect to mineral interactions and/or reaction processes in meteorites.

## 5. Applications

The technical development of analytical instrumentation will allow for the improved identification of organic compounds with more sensitivity compared to current analyses, and will advance comprehensive studies of the formation pathways and origins of extraterrestrial organic compounds. Furthermore, the new techniques will allow for the definitive identification of organic compounds in greatly reduced sample sizes (using micrograms of sample vs. current milligram requirements) under clean conditions to avoid terrestrial contamination, and will contribute to the successful analysis of the future sample-return mission from asteroid Ryugu by the Hayabusa2 spacecraft.

The Hayabusa2 spacecraft was launched on 3 December 2014, and arrived at asteroid 162173 Ryugu in August, 2018. The Ryugu asteroid has a surface with C-type reflectance spectra, suggesting that the asteroid surface is mainly composed of materials similar to that of carbonaceous chondrites, and the presence of hydrous minerals was confirmed by near-infrared spectroscopy (2.72 μm in wavelength) [23]. The first touch-and-go sampling was successfully performed on 22 February 2019, and the second sampling was also successful on 11 July 2019. The Hayabusa2 spacecraft will deliver the collected sample material to Earth in December, 2020.

The initial analysis of the Ryugu samples will focus on: (1) the detailed chemical and mineralogical characterization of the samples; (2) understanding the history of Ryugu and the Solar System in order to maximize the scientific achievement of the project; and (3) to prove the scientific potential of the samples to the community and demonstrate the analytical opportunities that are available [24]. A comprehensive study of soluble organic matter is planned by an international team for the initial analysis of the returned samples, in order to reveal the chemical evolution of Ryugu using high-sensitivity and high-resolution analytical methods as follows:

(1) High-resolution mass spectroscopy (HRMS) of various solvent extracts with electrospray ionization coupled with or without nano-liquid chromatography;
(2) DL-amino acid analysis using high-resolution column chromatography with high-sensitivity fluorescence spectroscopy coupled with HRMS; and
(3) In situ organic compound analysis and molecular imaging using DESI/HRMS.

## 6. Conclusions

New analytical techniques have been developed to study extraterrestrial organic compounds available in very limited quantities, especially for asteroidal materials returned by spacecraft. Clean room technology is essential to avoid terrestrial contamination and minimize the analytical background for the trace analysis. High-mass-resolution mass spectroscopy is very useful to definitively identify specific organic compounds from a very complex matrix of organic compounds. High-resolution and/or multi-dimensional chromatography can separate the complex organic compounds of structural and stereo isomers, and can reduce background signals. DESI/HRMS can provide in situ compound analysis with molecular mapping. These methods also provide high sensitivity, and sometimes non-destructive analysis, of these precious small and very-limited-quantity samples, such as the samples to be returned to Earth from the asteroid Ryugu by the Hayabusa2 spacecraft.
